# Supplementary material for: Understanding maternal sepsis risk factors and bacterial etiology: A case control study protocol
Source: PLoS One. 2024 Jun 26;19(6):e0305411. doi: 10.1371/journal.pone.0305411 (PMC11207175; doi:10.1371/journal.pone.0305411)
Supplement: S1 File — (DOCX) [file pone.0305411.s002.docx]

Supporting information: Understanding maternal sepsis risk factors and bacterial etiology: a case control study protocol

Kelly J Thompson^1,2^, Duy Pham Thanh^3^, Jane E Hirst^4^, Mark Woodward^1,4^, Hai Pham Thanh ^5^, Huong Tran Thi Lien ^5^, Kiet Tao Tuan ^5^, Binh Le Thanh ^5^, Evelyne Kestelyn^3^, Thuan Dang Trong^3^, Katie Harris^1^, Linh Nguyen Thi My^3^, Hien Vu Thi Minh^3^, Tuyen Ha Thanh^3^, Thanh Le Quang ^5^, Louise Thwaites^3^.

1. The George Institute for Global Health, University of New South Wales, Sydney, Australia
2. Nepean Blue Mountains Local Health District, Kingswood, Australia
3. Oxford University Clinical Research Unit, Ho Chi Minh City, Vietnam
4. The George Institute for Global Health, Imperial College London, London, United Kingdom
5. Tu Du Hospital, Ho Chi Minh City, Vietnam

**Corresponding Author:**

Dr Kelly Thompson, Research Fellow, Global Women’s Health

Organisation: The George Institute for Global Health

Telephone no.: + 61 451 307 097

Email: kthompson@georgeinstitute.org

| **Case Report Form** |
| --- |
| **Entry criteria for cases and controls (all must be YES from 1 to 6; and there is at least a “YES” answer in question 7 and 8 ):** |
| 1. Is the woman 18 years of age or older? 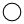 Yes 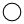 No 2. Is the woman ≥ 28 weeks gestation? 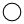 Yes 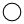 No 3. Does she have singleton birth? 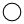 Yes 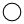 No 4. Woman is within the onset of labour / time of delivery to up to 7 days post-partum 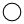 Yes 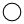 No 5. Is the woman SARS-CoV-2 **negative** (i.e. doesn’t have COVID symptoms such as   fever, flu like symptoms (Rapid Antigen Test required for those with COVID symptoms)? 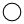 Yes 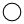 No   1. The woman **does not** have an uncomplicated, localised or chronic, infection 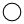 Yes 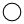 No *Including: Vaginosis, candidiasis, lower urinary tract infection, fungal infection of the skin, Otitis, Pharyngitis, Herpes simplex, Gonorrhoea, Syphilis, Trichomonas, Chlamydia, Hepatitis, HIV, Tuberculosis* 2. Suspected or confirmed infection with Obstetrically Modified quick SOFA 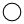 Yes 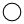 No (OM qSOFA) Score of ≥ 2?  \| Indicator \| Recorded value \| Mark \| \| --- \| --- \| --- \| \| 7.1. Systolic blood pressure (SBP) (mmHg) \|  \| 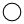 0 point 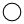 1 point \| \| 7.2. Respiratory Rate (breaths/mins) \|  \| 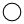 0 point 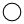 1 point \| \| 7.3 Altered mental status (GCS evaluation) \|  \| 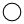 0 point 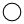 1 point \|   8. Follow-up with suspected maternal sepsis 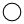 Yes 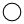 No  **9. Pre-screening evaluation 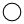 Satisfied 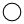 Not satisfied**  Người hoàn tất (tên viết tắt): Ngày hoàn tất: |
| **SCREENING SCR (1/2)** |
| Study Identifier Study Site Identifier Screening Number Subject Initials  **[34EN]** [ \| \| ] [ \| \| \| \| ] [ \| \| \| \| \| ] |
| Screening date [ \| ]/[ \| ]/[ \| ] (dd/mm/yy) |
| **1. Obstetrically Modified SOFA Score**  Document each of the d o m a i n s t o c a l c u l a t e e a c h c o m p o n e n t o f t h e Obstetrically Modified SOFA score using data for the most deranged (w o r s t) score within in the past 24 hours. To be eligible the patient must have an Obstetrically Modified SOFA score of 2 or more (see Data Dictionary).  **1.1. Respiratory** [ \| ] (0 – 2)  1.1.1. PaO_2_  [ \| \| ] mmHg  1.1.2. FiO_2_  [ \| \| ] %  1.1.3. PaO_2_/FiO_2_  [ \| \| ]    **1.2. Coagulation score** [ \| ] (0 – 2)  1.2.1. Platelets [ \| \| ] x 10^6^/ L  **1.3. Liver score** [ \| ] (0 – 2)  1.3.1. Total Bilirubin [ _ \| ] . [ ] µmol/L  **1.4.** **Cardiovascular score** [ \| ] (0 – 2)  1.4.1. Mean Arterial Pressure (MAP) [ \| \| ] mmHg  1.4.2. Vasopressors / Inotropes 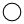 Có 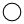 Không    **1.5. Renal score** [ \| ] (0 – 2)  1.5.1. Creatinine [_______] µmol/L  **1.6. CNS score** [ \| ] (0 – 2)  1.6.1. Alert or Rousable by  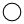 Normal  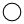 Voice  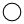 Pain  2. Intravenous antibiotics for suspected or confirmed infection 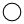 Yes 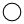 No  3. Requested cultures of any bodily fluid (blood, urine or CSF), including swab 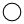 Yes 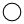 No  specimens (vaginal, rectal, nasopharyngeal, oropharyngeal) in women with suspected infection  **4. Screening result** 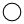 **Satisfied** 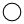 **Not satisfied**  Completed by (Initials): Date completed: |

| **GENERAL INFORMATION DEMO (1 / 1)** | | | |
| --- | --- | --- | --- |
| Study Identifier  **[34EN]** | Study Site Identifier  [ \| \| ] | Subject Identifier  [ \| \| \| \| ] | Subject Initials  [ \| \| \| \| \| ] |
| 1. Maternal age [ \| ] years 2. Gestational age (weeks and days) [ \| ] weeks [ \| ] days 3. Date of admission [ \| ]/[ \| ]/[ \| ] (dd/mm/yy) 4. Time of admission [ \| ]:[ \| ] (use 24 hour clock) 5. Maternal weight [ \| \| ] kgs 6. Maternal height [ \| \| ] cm 7. Number of pregnancies (including current or within the last 42 days) [ \|__] 8. Number of previous l iv e births (excluding current or within the last 42 days) [ \|__] 9. Number of previous s t il l births/neonatal deaths (excluding current or within the last 42 days) [ \|__] 10. Number of previous t e r m in a t io n s (excluding current or within the last 42 days) [ \|__] 11. Number of previous m is c a r r ia g e s (excluding current or within the last 42 days) [ \|__] 12. Total number of antenatal visits [ \|__] 13. Was this pregnancy an IVF pregnancy 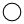 Yes 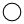No   Has the woman been vaccinated against the following medical conditions, and was this before or during the current pregnancy:   1. Influenza vaccination, before the pregnancy 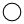 Yes 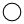No 2. Influenza vaccination, during the pregnancy 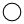 Yes 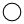No 3. Tetanus, before the pregnancy 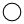 Yes 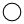No 4. Tetanus, during the pregnancy 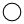 Yes 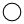No 5. COVID-19, before the pregnancy 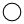 Yes 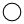No 6. COVID-19, during the pregnancy 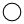 Yes 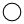No   Completed by (Initials): Date completed: | | | |

| **RELEVANT MEDICAL HISTORY MH (1 / 1)** | | | |
| --- | --- | --- | --- |
| Study Identifier  **[34EN]** | Study Site Identifier  [ \| \| ] | Subject Identifier  [ \| \| \| \| ] | Subject Initials  [ \| \| \| \| \| ] |
| **Please select any of the following that apply:**   1. Diabetes mellitus 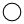 No 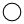 Type 1 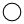 Type 2 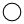 NA 2. Current gestational diabetes 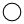 Yes 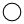 No 3. Past gestational diabetes 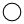 Yes 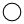 No 4. Aneamia (Hb<11g/dL) 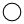 Yes 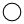 No 5. Essential hypertension 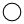 Yes 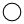 No 6. Past hypertensive disorder of pregnancy 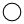 Yes 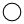 No 7. Current hypertensive disorder of pregnancy 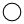 Yes 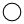 No   If Yes, Which of the following type of current hypertensive disorder of pregnancy? (refer to Data Dictionary for definitions)   - 1. Chronic hypertension 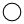 Yes 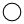 No   2. Gestational hypertension 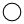 Yes 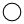 No   3. Preeclampsia 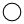 Yes 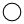 No   4. Severe Preeclampsia / HELLP Syndrome / Eclampsia 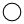 Yes 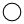 No  1. Renal disease 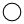 Yes 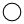 No 2. Thyroid disease 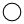 Yes 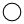 No 3. History of mental health disorder prior to pregnancy 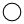 Yes 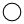 No   Details of mental health disorder and treatment ____________________________________________   1. History of mental health disorder during pregnancy 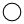 Yes 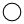 No   Details of mental health disorder and treatment ____________________________________________   1. Any infection during pregnancy that required treatment with antibiotics 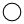 Yes 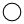 No   Details of type of infection and treatment ____________________________________________   1. Smoking status 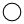 Current smoker 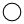 Ex-smoker 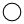 Never smoked 2. Drug and alcohol history   Alcohol in pregnancy 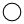 Yes 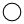 No If yes, cups per day [ \|__]  Illicit drugs in pregnancy 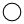 Yes 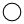 No  Completed by (Initials): Date completed: | | | |

| **COMPLICATIONS, LABOURS AND DELIVERY DETAIL DEV (1 / 1)** | | | | |
| --- | --- | --- | --- | --- |
| Study Identifier  **[34EN]** | Study Site Identifier  [ \| \| ] | Subject Identifier  [ \| \| \| \| ] | Subject Initials  [ \| \| \| \| \| | ] |
| 1. Any antenatal admissions to hospital 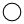 Yes 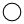 No   If Yes, provide details (reasons and gestational age) []   1. Threatened premature labour 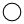 Yes 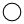 No   If Yes, provide details (times, equivalent gestational age) []   1. Preterm premature rupture of membranes 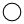 Yes 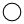 No   If Yes, provide details (gestational age) []   1. Antepartum haemorrhage 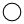 Yes 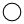 No   4.1. Reason(s): 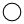 Uterine phlegmon 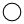 Genital lesions  Placental omission Blood clotting disorders  Other reason(s) []  4.2. Total Loss Blood Volume: [__________] ml  4.3. Postpartum haemorrhage > 1000mLs Yes No     1. Fetal anomaly Yes No   If Yes, provide details [ ]   1. Fetal growth restriction Yes No   If Yes, provide details [ ]   1. Mode of delivery Planned elective LSCS cesarean section in labor LSCS   Spontaneous delivery Operative delivery   1. Labour onset Spontaneous Induction Planned elective LSCS Emergency LSCS 2. Pain relief in labour Yes No   If Yes, provide details (name of drug) []   1. Medications in labour and delivery Yes No   10.1. Specify: : Oxytocin Ergometrin Acid Tranexamic  Duratocin Lidocain  Other: [ ]   1. Antibiotics in labour Yes No   If Yes, provide details (name of antibiotics) []   1. Placental abruption Yes No 2. Duration of rupture of membranes in hours _____ 3. Shoulder dystocia Yes No NA 4. 3rd or 4th degree perineal tear Yes No NA 5. Manual removal of placenta Yes No NA   Completed by (Initials): Date completed: | | | | |

| **IDENTIFICATION OF INFECTION/SEPSIS & TREATMENT (CASE ONLY) TREAT (1 / 1)** |
| --- |
| Study Identifier Study Site Identifier Subject Identifier Subject Initials  **[34EN]** [ \| \| ] [ \| \| \| \| ] [ \| \| \| \| \| ] |
| **CLINICAL IDENTIFICATION OF INFECTION / SEPSIS AND TREATMENT (CASES ONLY)**   1. Date of suspected or confirmed infection [ \| ]/[ \| ]/[ \| ] (dd/mm/yy)   INFECTION SITES   1. **First** defined source of infection: [ ] 2. How was the source of infection: Clinical identification Lab based confirmation identified   3.1. If culture taken, source of:  Blood Urine CSF culture Vaginal swab Rectal swab Nasopharyngeal swab Oropharyngeal swab Abdominal fluid Lochia  Other, specify: [ ]   1. Organism identified [ ] 2. Date diagnosed [ \| ]/[ \| ]/[ \| ] (dd/mm/yy) 3. **Second** defined source of infection: [ ] 4. How was the source of infection: Clinical identification Lab based confirmation identified   7.1. If culture taken, source of:  Blood Urine CSF culture Vaginal swab Rectal swab Nasopharyngeal swab Oropharyngeal swab Abdominal fluid Lochia  Other, specify: [ ]   1. Organism identified [ ] 2. Date diagnosed [ \| ]/[ \| ]/[ \| ] (dd/mm/yy) 3. **Third** defined source of infection: [ ] 4. How was the source of infection: Clinical identification Lab based confirmation identified   11.1. If culture taken, source of:  Blood Urine CSF culture Vaginal swab Rectal swab Nasopharyngeal swab Oropharyngeal swab Abdominal fluid Lochia  Other, specify: [ ]   1. Organism identified [ ] 2. Date diagnosed [ \| ]/[ \| ]/[ \| ] (dd/mm/yy) 3. Was the patient admitted to ICU / HDU Yes No   Completed by (Initials): Date completed: |

**34EN – Understanding maternal infection and sepsis in Tu Du Hospital**

| **ANTIMICROBIALS ABS (1 / 1)** | | | | | | | | | |
| --- | --- | --- | --- | --- | --- | --- | --- | --- | --- |
| Study Identifier  **[34EN]** | Study Site Identifier  [ \| \| ] |  |  | Subject Identifier  [ \| \| \| \| ] |  | Subject Initials  [ \| \| \| \| \| ] |  |  |  |
| \| **Name of antimicrobial** \| **Dose** \| **Frequency**  **(times per day)** \| **Start date (dd/mm/yy)** \| **Stop date**  **(dd/mm/yy)** \| **Route** \| **Total no. of doses given** \| \| --- \| --- \| --- \| --- \| --- \| --- \| --- \| \|  \|  \|  \|  \|  \|  \|  \| \|  \|  \|  \|  \|  \|  \|  \| \|  \|  \|  \|  \|  \|  \|  \| \|  \|  \|  \|  \|  \|  \|  \| \|  \|  \|  \|  \|  \|  \|  \| \|  \|  \|  \|  \|  \|  \|  \| \|  \|  \|  \|  \|  \|  \|  \| \|  \|  \|  \|  \|  \|  \|  \| \|  \|  \|  \|  \|  \|  \|  \|   Completed by (Initials): Date completed: | | | | | | | | | |

CRF V2.1 EN 26Jul23 **(8 / 10)**

| **OUTCOME** |  |  | **FINAL (1 / 1)** | |
| --- | --- | --- | --- | --- |
| Study Identifier  **[34EN]** | Study Site Identifier  [ \| \| ] | Subject Identifier  [ \| \| \| \| ] | Subject Initials  [ \| \| \| \| \| | ] |
| **MATERNAL LENGTH OF STAY OUTCOMES**   1. Date of hospital discharge [ \| ]/[ \| ]/[ \| ] (dd/mm/yy) 2. Time of hospital discharge [ \| ]:[ \| ] (24 hour) 3. Vital status at discharge Discharge, recover Die 4. Discharge destination: home transfer to other hospital   **NEONATAL OUTCOMES & COMPLICATIONS**  1. Date of Birth [ \| ]/[ \| ]/[ \| ] (dd/mm/yy)  2. Time of birth [ \| ]:[ \| ] (use 24 hour clock)  3. APGAR Score (5-min) [ \| ]  4. Mode of birth Vaginal spontaneous Vaginal assisted (forceps / vaccum)  Caesarean section  5.Birth weight [ \| \| \| ] (grams)  6. Birth length [ \| ] (cms)  7. Head circumference [ \| ] (cms)  8. Preterm delivery <37 weeks Yes No  9. Neonatal hypoglycaemia BGL <2.6mmol/L Yes No  10. Prolonged use (>4 hours) of continuous positive airway pressure Yes No  11. Intubation Yes No  12. Hypoxic ischaemic encephalopathy Yes No  13. Neonatal Intensive Care Unit or Special Care Nursery admission at <4 Yes No  13.1. in ICU ≥ 24h Yes No  14. Advanced resuscitation of baby after birth Yes No  15. Cord arterial gas pH <7.05 pH ≥7.05 didn’t perform  16. Stillbirth Yes No  17. Perinatal death Yes No  Completed by (Initials): Date completed: | | | | |

| **HO S P I T A L ADMIISSION DETAILS HOS_ADMIT (1 / 1)** | | | | |
| --- | --- | --- | --- | --- |
| Study Identifier  **[34EN]** | Study Site Identifier  [ \| \| ] | Subject Identifier  [ \| \| \| \| ] | Subject Initials  [ \| \| \| \| \| | ] |

| **ICU ADMISSION DETAILS AND ORGAN SUPPORTIVE THERAPY**   1. Date of I C U admission [ \| ]/[ \| ]/[ \| ] (dd/mm/yy) 2. Date of ICU discharge [ \| ]/[ \| ]/[ \| ] (dd/mm/yy) 3. Treatment with inotropes / vasopressors Yes No   If Yes, Start date [ \| ]/[ \| ]/[ \| ] Stop date [ \| ]/[ \| ]/[ \| ] (dd/mm/yy)   1. Treatment with mechanical ventilation Yes No unknow   If Yes, Start date [ \| ]/[ \| ]/[ \| ] Stop date [ \| ]/[ \| ]/[ \| ] (dd/mm/yy)   1. Treatment with renal replacement therapy Yes No unknow   ( Continuous dialysis, hemodialysis, peritoneal osmolation)  If Yes, Start date [ \| ]/[ \| ]/[ \| ] Stop date [ \| ]/[ \| ]/[ \| ] (dd/mm/yy)  Completed by (Initials): Date completed: |
| --- |

| **LAB TEST RESULTS LAB (1 / 8)** |
| --- |
| Study Identifier Study Site Identifier Patient Number Patient initials  [34EN] [102] [__\|__\|__\|__\|__] [__\|__\|__\|__\|__] |
| Date of receipt of sample [___\|___]/[___\|___]/[___\|___\|___\|___] (dd/mm/yyyy)  Case ⭘ Sepsis ⭘ Infection ⭘ Control |
| **GRAM POSITIVE BACTERIA** |
| \| 1. Sample 1 \| ⭘ Vaginal swab  ⭘ Lochia \| ⭘ Rectal swab  ⭘ Urine \| ⭘ Blood  ⭘ Other[………………….] \| \| \| --- \| --- \| --- \| --- \| --- \| \| 1. Isolation 1 \| ⭘Streptococcus  group A \| ⭘Streptococcus group B \| ⭘Streptococcus  group C \| \| \| ⭘Streptococcus  group G \| ⭘Staphylococcus aureus \| ⭘Enterococcus spp \| \| \| ⭘ Neg \| ⭘Not done \| ⭘ Other [………………..] \| \| \| 1. Name of Isolation 1 \| […………………………………………….] \| \| \| \| \| \| 1. Date of isolation 1 \| [___\|___]/[___\|___]/[___\|___\|___\|___] (dd/mm/yyyy) \| \| \| \| \| \| 1. Susceptibitity Erythromycin 1 \| ⭘ S \| ⭘ I \| ⭘ R \| ⭘ Not done \| \| 1. MIC Erythromycin 1 \| […………………………..] \| \| \| \| \| \| 1. Susceptibitity Trimethoprim-Sulfamethaxazole 1 \| ⭘ S \| ⭘ I \| ⭘ R \| ⭘ Not done \| \| 1. MIC Trimethoprim-Sulfamethaxazole 1 \| […………………………..] \| \| \| \| \| \| 1. SusceptibilityLevofloxacin 1 \| ⭘ S \| ⭘ I \| ⭘ R \| ⭘ Not done \| \| 1. MIC Levofloxacin 1 \| […………………………..] \| \| \| \| \| \| 1. SusceptibilityMeropenem 1 \| ⭘ S \| ⭘ I \| ⭘ R \| ⭘ Not done \| \| 1. MIC Meropenem 1 \| […………………………..] \| \| \| \| \| \| 1. SusceptibilityVancomycin 1 \| ⭘ S \| ⭘ I \| ⭘ R \| ⭘ Not done \| \| 1. MIC Vancomycin 1 \| […………………………..] \| \| \| \| \| \| 1. SusceptibilityClindamycin 1 \| ⭘ S \| ⭘ I \| ⭘ R \| ⭘ Not done \| \| 1. MIC Clindamycin 1 \| […………………………..] \| \| \| \| \| \| 1. Susceptibitity Cefotaxime 1 \| ⭘ S \| ⭘ I \| ⭘ R \| ⭘ Not done \| \| 1. MIC Cefotaxime 1 \| […………………………..] \| \| \| \| \| \| 1. SusceptibilityMoxifloxacin 1 \| ⭘ S \| ⭘ I \| ⭘ R \| ⭘ Not done \| \| 1. MIC Moxifloxacin 1 \| […………………………..] \| \| \| \| \| \| 1. SusceptibilityTetracycline 1 \| ⭘ S \| ⭘ I \| ⭘ R \| ⭘ Not done \| \| 1. MIC Tetracycline 1 \| […………………………..] \| \| \| \| \| \| 1. SusceptibilityChloramphenicol 1 \| ⭘ S \| ⭘ I \| ⭘ R \| ⭘ Not done \| \| 1. MIC Chloramphenicol 1 \| […………………………..] \| \| \| \| \| \| 1. Susceptibitity Amoxicillin 1 \| ⭘ S \| ⭘ I \| ⭘ R \| ⭘ Not done \| \| 1. MIC Amoxicillin 1 \| […………………………..] \| \| \| \| \| \| 1. SusceptibilityLinezolid 1 \| ⭘ S \| ⭘ I \| ⭘ R \| ⭘ Not done \| \| 1. MIC Linezolid 1 \| […………………………..] \| \| \| \| \| \| 1. SusceptibilityCefepime 1 \| ⭘ S \| ⭘ I \| ⭘ R \| ⭘ Not done \| \| 1. MIC Cefepime 1 \| […………………………..] \| \| \| \| \| \| 1. SusceptibilityAzithromycin 1 \| ⭘ S \| ⭘ I \| ⭘ R \| ⭘ Not done \| \| 1. MIC Azithromycin 1 \| […………………………..] \| \| \| \| \| \| 1. Susceptibitity Clarithromycin 1 \| ⭘ S \| ⭘ I \| ⭘ R \| ⭘ Not done \| \| 1. MIC Clarithromycin 1 \| […………………………..] \| \| \| \| \| 1. SusceptibilityPenicillin G 1 \| ⭘ S \| ⭘ I \| ⭘ R \| ⭘ Not done \| \| 1. MIC Penicillin G 1 \| […………………………..] \| \| \| \| \| 1. SusceptibilityOxacillin 1 \| ⭘ S \| ⭘ I \| ⭘ R \| ⭘ Not done \| \| 1. MIC Oxacillin 1 \| […………………………..] \| \| \| \| \| \| 1. SusceptibilityDoxycycline 1 \| ⭘ S \| ⭘ I \| ⭘ R \| ⭘ Not done \| \| 1. MIC Doxycycline 1 \| […………………………..] \| \| \| \| \| \| 1. SusceptibilityDaptomycin 1 \| ⭘ S \| ⭘ I \| ⭘ R \| ⭘ Not done \| \| 1. MIC Daptomycin 1 \| […………………………..] \| \| \| \| \| \| 1. SusceptibilityMinocycline 1 \| ⭘ S \| ⭘ I \| ⭘ R \| ⭘ Not done \| \| 1. MIC Minocycline 1 \| […………………………..] \| \| \| \| \| \| 1. SusceptibilityCiprofloxacin 1 \| ⭘ S \| ⭘ I \| ⭘ R \| ⭘ Not done \| \| 1. MIC Ciprofloxacin 1 \| […………………………..] \| \| \| \| \| \| 1. SusceptibilityGentamicin 1 \| ⭘ S \| ⭘ I \| ⭘ R \| ⭘ Not done \| \| 1. MIC Gentamicin 1 \| […………………………..] \| \| \| \| \| \| 1. Susceptibitity Ampicillin 1 \| ⭘ S \| ⭘ I \| ⭘ R \| ⭘ Not done \| \| 1. MIC Ampicillin 1 \| […………………………..] \| \| \| \| \| \| 1. SusceptibilityGentmicin-Syn 1 \| ⭘ S \| ⭘ I \| ⭘ R \| ⭘ Not done \| \| 1. MIC Gentmicin-Syn 1 \| […………………………..] \| \| \| \| \| \| 1. SusceptibilityNitrofurantoin 1 \| ⭘ S \| ⭘ I \| ⭘ R \| ⭘ Not done \| \| 1. MIC Nitrofurantoin 1 \| […………………………..] \| \| \| \| \| |

| **LAB TEST RESULTS LAB (2 / 8)** |
| --- |
| \| 1. Sample 2 \| \| ⭘ Vaginal swab  ⭘ Lochia \| \| ⭘ Rectal swab  ⭘ Urine \| \| ⭘ Blood  ⭘ Other [………………….] \| \| \| \| --- \| --- \| --- \| --- \| --- \| --- \| --- \| --- \| --- \| \| 1. Isolation 2 \| \| ⭘Streptococcus  group A \| \| ⭘Streptococcus group B \| \| ⭘Streptococcus  group C \| \| \| \| ⭘Streptococcus  group G \| \| ⭘Staphylococcus aureus \| \| ⭘Enterococcus spp \| \| \| \| ⭘ NEG \| \| ⭘Not done \| \| ⭘ Other [………………..] \| \| \| \| 1. Name of isolation 2 \| \| […………………………………………….] \| \| \| \| \| \| \| \| 1. Date of isolation 2 \| \| [___\|___]/[___\|___]/[___\|___\|___\|___] (dd/mm/yyyy) \| \| \| \| \| \| \| \| 1. Susceptibitity Erythromycin 2 \| \| ⭘ S \| \| ⭘ I \| \| ⭘ R \| \| ⭘ Not done \| \| 1. MIC Erythromycin 2 \| \| […………………………..] \| \| \| \| \| \| \| \| 1. Susceptibitity Trimethoprim-Sulfamethaxazole 2 \| \| ⭘ S \| \| ⭘ I \| \| ⭘ R \| \| ⭘ Not done \| \| 1. MIC Trimethoprim-Sulfamethaxazole 2 \| \| […………………………..] \| \| \| \| \| \| \| \| 1. SusceptibilityLevofloxacin 2 \| \| ⭘ S \| \| ⭘ I \| \| ⭘ R \| \| ⭘ Not done \| \| 1. MIC Levofloxacin 2 \| \| […………………………..] \| \| \| \| \| \| \| \| 1. SusceptibilityMeropenem 2 \| \| ⭘ S \| \| ⭘ I \| \| ⭘ R \| \| ⭘ Not done \| \| 1. MIC Meropenem 2 \| \| […………………………..] \| \| \| \| \| \| \| \| 1. SusceptibilityVancomycin 2 \| \| ⭘ S \| \| ⭘ I \| \| ⭘ R \| \| ⭘ Not done \| \| 1. MIC Vancomycin 2 \| \| […………………………..] \| \| \| \| \| \| \| \| 1. SusceptibilityClindamycin 2 \| \| ⭘ S \| \| ⭘ I \| \| ⭘ R \| \| ⭘ Not done \| \| 1. MIC Clindamycin 2 \| \| […………………………..] \| \| \| \| \| \| \| \| 1. Susceptibitity Cefotaxime 2 \| \| ⭘ S \| \| ⭘ I \| \| ⭘ R \| \| ⭘ Not done \| \| 1. MIC Cefotaxime 2 \| \| […………………………..] \| \| \| \| \| \| \| \| 1. SusceptibilityMoxifloxacin 2 \| \| ⭘ S \| \| ⭘ I \| \| ⭘ R \| \| ⭘ Not done \| \| 1. MIC Moxifloxacin 2 \| \| […………………………..] \| \| \| \| \| \| \| \| 1. SusceptibilityTetracycline 2 \| \| ⭘ S \| \| ⭘ I \| \| ⭘ R \| \| ⭘ Not done \| \| 1. MIC Tetracycline 2 \| […………………………..] \| \| \| \| \| \| \| \| \| 1. SusceptibilityChloramphenicol 2 \| ⭘ S \| \| ⭘ I \| \| ⭘ R \| \| ⭘ Not done \| \| \| 1. MIC Chloramphenicol 2 \| […………………………..] \| \| \| \| \| \| \| \| \| 1. Susceptibitity Amoxicillin 2 \| ⭘ S \| \| ⭘ I \| \| ⭘ R \| \| ⭘ Not done \| \| \| 1. MIC Amoxicillin 2 \| […………………………..] \| \| \| \| \| \| \| \| \| 1. SusceptibilityLinezolid 2 \| ⭘ S \| \| ⭘ I \| \| ⭘ R \| \| ⭘ Not done \| \| \| 1. MIC Linezolid 2 \| […………………………..] \| \| \| \| \| \| \| \| \| 1. SusceptibilityCefepime 2 \| ⭘ S \| \| ⭘ I \| \| ⭘ R \| \| ⭘ Not done \| \| \| 1. MIC Cefepime 2 \| […………………………..] \| \| \| \| \| \| \| \| \| 1. SusceptibilityAzithromycin 2 \| ⭘ S \| \| ⭘ I \| \| ⭘ R \| \| ⭘ Not done \| \| \| 1. MIC Azithromycin 2 \| […………………………..] \| \| \| \| \| \| \| \| \| 1. Susceptibitity Clarithromycin 2 \| ⭘ S \| \| ⭘ I \| \| ⭘ R \| \| ⭘ Not done \| \| \| 1. MIC Clarithromycin 2 \| […………………………..] \| \| \| \| \| \| \| \| \| 1. SusceptibilityPenicillin G 2 \| ⭘ S \| \| ⭘ I \| \| ⭘ R \| \| ⭘ Not done \| \| \| 1. MIC Penicillin G 2 \| […………………………..] \| \| \| \| \| \| \| \| \| 1. SusceptibilityOxacillin 2 \| ⭘ S \| \| ⭘ I \| \| ⭘ R \| \| ⭘ Not done \| \| \| 1. MIC Oxacillin 2 \| […………………………..] \| \| \| \| \| \| \| \| \| 1. SusceptibilityDoxycycline 2 \| ⭘ S \| \| ⭘ I \| \| ⭘ R \| \| ⭘ Not done \| \| \| 1. MIC Doxycycline 2 \| […………………………..] \| \| \| \| \| \| \| \| \| 1. SusceptibilityDaptomycin 2 \| ⭘ S \| \| ⭘ I \| \| ⭘ R \| \| ⭘ Not done \| \| \| 1. MIC Daptomycin 2 \| […………………………..] \| \| \| \| \| \| \| \| \| 1. SusceptibilityMinocycline 2 \| ⭘ S \| \| ⭘ I \| \| ⭘ R \| \| ⭘ Not done \| \| \| 1. MIC Minocycline 2 \| […………………………..] \| \| \| \| \| \| \| \| \| 1. SusceptibilityCiprofloxacin 2 \| ⭘ S \| \| ⭘ I \| \| ⭘ R \| \| ⭘ Not done \| \| \| 1. MIC Ciprofloxacin 2 \| […………………………..] \| \| \| \| \| \| \| \| \| 1. SusceptibilityGentamicin 2 \| ⭘ S \| \| ⭘ I \| \| ⭘ R \| \| ⭘ Not done \| \| \| 1. MIC Gentamicin 2 \| […………………………..] \| \| \| \| \| \| \| \| \| 1. Susceptibitity Ampicillin 2 \| ⭘ S \| \| ⭘ I \| \| ⭘ R \| \| ⭘ Not done \| \| \| 1. MIC Ampicillin 2 \| […………………………..] \| \| \| \| \| \| \| \| \| 1. SusceptibilityGentmicin-Syn 2 \| ⭘ S \| \| ⭘ I \| \| ⭘ R \| \| ⭘ Not done \| \| \| 1. MIC Gentmicin-Syn 2 \| […………………………..] \| \| \| \| \| \| \| \| \| 1. SusceptibilityNitrofurantoin 2 \| ⭘ S \| \| ⭘ I \| \| ⭘ R \| \| ⭘ Not done \| \| \| 1. MIC Nitrofurantoin 2 \| […………………………..] \| \| \| \| \| \| \| \|  \| **GRAM POSITIVE BACTERIA** \| \| \| \| \| \| \| \| \| \| --- \| --- \| --- \| --- \| --- \| --- \| --- \| --- \| --- \| \| 1. Sample 3 \| \| ⭘ Vaginal swab  ⭘ Lochia \| \| ⭘ Rectal swab  ⭘ Urine \| \| ⭘ Blood  ⭘ Other [………………….] \| \| \| \| 1. Isolation 3 \| \| ⭘Streptococcus  group A \| \| ⭘Streptococcus group B \| \| ⭘Streptococcus  group C \| \| \| \| ⭘Streptococcus  group G \| \| ⭘Staphylococcus aureus \| \| ⭘Enterococcus spp \| \| \| \| ⭘ NEG \| \| ⭘Not done \| \| ⭘ Other [………………..] \| \| \| \| 1. Name of isolation 3 \| \| […………………………………………….] \| \| \| \| \| \| \| \| 1. Date of isolation 3 \| \| [___\|___]/[___\|___]/[___\|___\|___\|___] (dd/mm/yyyy) \| \| \| \| \| \| \| \| 1. Susceptibitity Erythromycin 3 \| \| ⭘ S \| \| ⭘ I \| \| ⭘ R \| \| ⭘ Not done \| \| 1. MIC Erythromycin 3 \| \| […………………………..] \| \| \| \| \| \| \| \| 1. Susceptibitity Trimethoprim-Sulfamethaxazole 3 \| \| ⭘ S \| \| ⭘ I \| \| ⭘ R \| \| ⭘ Not done \| \| 1. MIC Trimethoprim-Sulfamethaxazole 3 \| \| […………………………..] \| \| \| \| \| \| \| \| 1. SusceptibilityLevofloxacin 3 \| \| ⭘ S \| \| ⭘ I \| \| ⭘ R \| \| ⭘ Not done \| \| 1. MIC Levofloxacin 3 \| \| […………………………..] \| \| \| \| \| \| \| \| 1. SusceptibilityMeropenem 3 \| \| ⭘ S \| \| ⭘ I \| \| ⭘ R \| \| ⭘ Not done \| \| 1. MIC Meropenem 3 \| \| […………………………..] \| \| \| \| \| \| \| \| 1. SusceptibilityVancomycin 3 \| \| ⭘ S \| \| ⭘ I \| \| ⭘ R \| \| ⭘ Not done \| \| 1. MIC Vancomycin 3 \| \| […………………………..] \| \| \| \| \| \| \| \| 1. SusceptibilityClindamycin 3 \| \| ⭘ S \| \| ⭘ I \| \| ⭘ R \| \| ⭘ Not done \| \| 1. MIC Clindamycin 3 \| \| […………………………..] \| \| \| \| \| \| \| \| 1. Susceptibitity Cefotaxime 3 \| \| ⭘ S \| \| ⭘ I \| \| ⭘ R \| \| ⭘ Not done \| \| 1. MIC Cefotaxime 3 \| \| […………………………..] \| \| \| \| \| \| \| \| 1. SusceptibilityMoxifloxacin 3 \| \| ⭘ S \| \| ⭘ I \| \| ⭘ R \| \| ⭘ Not done \| \| 1. MIC Moxifloxacin 3 \| \| […………………………..] \| \| \| \| \| \| \| \| 1. SusceptibilityTetracycline 3 \| \| ⭘ S \| \| ⭘ I \| \| ⭘ R \| \| ⭘ Not done \| \| 1. MIC Tetracycline 3 \| […………………………..] \| \| \| \| \| \| \| \| \| 1. SusceptibilityChloramphenicol 3 \| ⭘ S \| \| ⭘ I \| \| ⭘ R \| \| ⭘ Not done \| \| \| 1. MIC Chloramphenicol 3 \| […………………………..] \| \| \| \| \| \| \| \| \| 1. Susceptibitity Amoxicillin 3 \| ⭘ S \| \| ⭘ I \| \| ⭘ R \| \| ⭘ Not done \| \| \| 1. MIC Amoxicillin 3 \| […………………………..] \| \| \| \| \| \| \| \| \| 1. SusceptibilityLinezolid 3 \| ⭘ S \| \| ⭘ I \| \| ⭘ R \| \| ⭘ Not done \| \| \| 1. MIC Linezolid 3 \| […………………………..] \| \| \| \| \| \| \| \| \| 1. SusceptibilityCefepime 3 \| ⭘ S \| \| ⭘ I \| \| ⭘ R \| \| ⭘ Not done \| \| \| 1. MIC Cefepime 3 \| […………………………..] \| \| \| \| \| \| \| \| \| 1. SusceptibilityAzithromycin 3 \| ⭘ S \| \| ⭘ I \| \| ⭘ R \| \| ⭘ Not done \| \| \| 1. MIC Azithromycin 3 \| […………………………..] \| \| \| \| \| \| \| \| \| 1. Susceptibitity Clarithromycin 3 \| ⭘ S \| \| ⭘ I \| \| ⭘ R \| \| ⭘ Not done \| \| \| 1. MIC Clarithromycin 3 \| […………………………..] \| \| \| \| \| \| \| \| \| 1. SusceptibilityPenicillin G 3 \| ⭘ S \| \| ⭘ I \| \| ⭘ R \| \| ⭘ Not done \| \| \| 1. MIC Penicillin G 3 \| […………………………..] \| \| \| \| \| \| \| \| \| 1. SusceptibilityOxacillin 3 \| ⭘ S \| \| ⭘ I \| \| ⭘ R \| \| ⭘ Not done \| \| \| 1. MIC Oxacillin 3 \| […………………………..] \| \| \| \| \| \| \| \| \| 1. SusceptibilityDoxycycline 3 \| ⭘ S \| \| ⭘ I \| \| ⭘ R \| \| ⭘ Not done \| \| \| 1. MIC Doxycycline 3 \| […………………………..] \| \| \| \| \| \| \| \| \| 1. SusceptibilityDaptomycin 3 \| ⭘ S \| \| ⭘ I \| \| ⭘ R \| \| ⭘ Not done \| \| \| 1. MIC Daptomycin 3 \| […………………………..] \| \| \| \| \| \| \| \| \| 1. SusceptibilityMinocycline 3 \| ⭘ S \| \| ⭘ I \| \| ⭘ R \| \| ⭘ Not done \| \| \| 1. MIC Minocycline 3 \| […………………………..] \| \| \| \| \| \| \| \| \| 1. SusceptibilityCiprofloxacin 3 \| ⭘ S \| \| ⭘ I \| \| ⭘ R \| \| ⭘ Not done \| \| \| 1. MIC Ciprofloxacin 3 \| […………………………..] \| \| \| \| \| \| \| \| \| 1. SusceptibilityGentamicin 3 \| ⭘ S \| \| ⭘ I \| \| ⭘ R \| \| ⭘ Not done \| \| \| 1. MIC Gentamicin 3 \| […………………………..] \| \| \| \| \| \| \| \| \| 1. Susceptibitity Ampicillin 3 \| ⭘ S \| \| ⭘ I \| \| ⭘ R \| \| ⭘ Not done \| \| \| 1. MIC Ampicillin 3 \| […………………………..] \| \| \| \| \| \| \| \| \| 1. SusceptibilityGentmicin-Syn 3 \| ⭘ S \| \| ⭘ I \| \| ⭘ R \| \| ⭘ Not done \| \| \| 1. MIC Gentmicin-Syn 3 \| […………………………..] \| \| \| \| \| \| \| \| \| 1. SusceptibilityNitrofurantoin 3 \| ⭘ S \| \| ⭘ I \| \| ⭘ R \| \| ⭘ Not done \| \| \| 1. MIC Nitrofurantoin 3 \| […………………………..] \| \| \| \| \| \| \| \|  \| **GRAM POSITIVE BACTERIA** \| \| \| \| \| \| \| \| \| \| --- \| --- \| --- \| --- \| --- \| --- \| --- \| --- \| --- \| \| 1. Sample 4 \| \| ⭘ Vaginal swab  ⭘ Lochia \| \| ⭘ Rectal swab  ⭘ Urine \| \| ⭘ Blood  ⭘ Other [………………….] \| \| \| \| 1. Isolation 4 \| \| ⭘Streptococcus  group A \| \| ⭘Streptococcus group B \| \| ⭘Streptococcus  group C \| \| \| \| ⭘Streptococcus  group G \| \| ⭘Staphylococcus aureus \| \| ⭘Enterococcus spp \| \| \| \| ⭘ NEG \| \| ⭘Not done \| \| ⭘ Other [………………..] \| \| \| \| 1. Name of isolation 4 \| \| […………………………………………….] \| \| \| \| \| \| \| \| 1. Date of isolation 4 \| \| [___\|___]/[___\|___]/[___\|___\|___\|___] (dd/mm/yyyy) \| \| \| \| \| \| \| \| 1. Susceptibitity Erythromycin 4 \| \| ⭘ S \| \| ⭘ I \| \| ⭘ R \| \| ⭘ Not done \| \| 1. MIC Erythromycin 4 \| \| […………………………..] \| \| \| \| \| \| \| \| 1. Susceptibitity Trimethoprim-Sulfamethaxazole 4 \| \| ⭘ S \| \| ⭘ I \| \| ⭘ R \| \| ⭘ Not done \| \| 1. MIC Trimethoprim-Sulfamethaxazole 4 \| \| […………………………..] \| \| \| \| \| \| \| \| 1. SusceptibilityLevofloxacin 4 \| \| ⭘ S \| \| ⭘ I \| \| ⭘ R \| \| ⭘ Not done \| \| 1. MIC Levofloxacin 4 \| \| […………………………..] \| \| \| \| \| \| \| \| 1. SusceptibilityMeropenem 4 \| \| ⭘ S \| \| ⭘ I \| \| ⭘ R \| \| ⭘ Not done \| \| 1. MIC Meropenem 4 \| \| […………………………..] \| \| \| \| \| \| \| \| 1. SusceptibilityVancomycin 4 \| \| ⭘ S \| \| ⭘ I \| \| ⭘ R \| \| ⭘ Not done \| \| 1. MIC Vancomycin 4 \| \| […………………………..] \| \| \| \| \| \| \| \| 1. SusceptibilityClindamycin 4 \| \| ⭘ S \| \| ⭘ I \| \| ⭘ R \| \| ⭘ Not done \| \| 1. MIC Clindamycin 4 \| \| […………………………..] \| \| \| \| \| \| \| \| 1. Susceptibitity Cefotaxime 4 \| \| ⭘ S \| \| ⭘ I \| \| ⭘ R \| \| ⭘ Not done \| \| 1. MIC Cefotaxime 4 \| \| […………………………..] \| \| \| \| \| \| \| \| 1. SusceptibilityMoxifloxacin 4 \| \| ⭘ S \| \| ⭘ I \| \| ⭘ R \| \| ⭘ Not done \| \| 1. MIC Moxifloxacin 4 \| \| […………………………..] \| \| \| \| \| \| \| \| 1. SusceptibilityTetracycline 4 \| \| ⭘ S \| \| ⭘ I \| \| ⭘ R \| \| ⭘ Not done \| \| 1. MIC Tetracycline 4 \| […………………………..] \| \| \| \| \| \| \| \| \| 1. SusceptibilityChloramphenicol 4 \| ⭘ S \| \| ⭘ I \| \| ⭘ R \| \| ⭘ Not done \| \| \| 1. MIC Chloramphenicol 4 \| […………………………..] \| \| \| \| \| \| \| \| \| 1. Susceptibitity Amoxicillin 4 \| ⭘ S \| \| ⭘ I \| \| ⭘ R \| \| ⭘ Not done \| \| \| 1. MIC Amoxicillin 4 \| […………………………..] \| \| \| \| \| \| \| \| \| 1. SusceptibilityLinezolid 4 \| ⭘ S \| \| ⭘ I \| \| ⭘ R \| \| ⭘ Not done \| \| \| 1. MIC Linezolid 4 \| […………………………..] \| \| \| \| \| \| \| \| \| 1. SusceptibilityCefepime 4 \| ⭘ S \| \| ⭘ I \| \| ⭘ R \| \| ⭘ Not done \| \| \| 1. MIC Cefepime 4 \| […………………………..] \| \| \| \| \| \| \| \| \| 1. SusceptibilityAzithromycin 4 \| ⭘ S \| \| ⭘ I \| \| ⭘ R \| \| ⭘ Not done \| \| \| 1. MIC Azithromycin 4 \| […………………………..] \| \| \| \| \| \| \| \| \| 1. Susceptibitity Clarithromycin 4 \| ⭘ S \| \| ⭘ I \| \| ⭘ R \| \| ⭘ Not done \| \| \| 1. MIC Clarithromycin 4 \| […………………………..] \| \| \| \| \| \| \| \| \| 1. SusceptibilityPenicillin G 4 \| ⭘ S \| \| ⭘ I \| \| ⭘ R \| \| ⭘ Not done \| \| \| 1. MIC Penicillin G 4 \| […………………………..] \| \| \| \| \| \| \| \| \| 1. SusceptibilityOxacillin 4 \| ⭘ S \| \| ⭘ I \| \| ⭘ R \| \| ⭘ Not done \| \| \| 1. MIC Oxacillin 4 \| […………………………..] \| \| \| \| \| \| \| \| \| 1. SusceptibilityDoxycycline 4 \| ⭘ S \| \| ⭘ I \| \| ⭘ R \| \| ⭘ Not done \| \| \| 1. MIC Doxycycline 4 \| […………………………..] \| \| \| \| \| \| \| \| \| 1. SusceptibilityDaptomycin 4 \| ⭘ S \| \| ⭘ I \| \| ⭘ R \| \| ⭘ Not done \| \| \| 1. MIC Daptomycin 4 \| […………………………..] \| \| \| \| \| \| \| \| \| 1. SusceptibilityMinocycline 4 \| ⭘ S \| \| ⭘ I \| \| ⭘ R \| \| ⭘ Not done \| \| \| 1. MIC Minocycline 4 \| […………………………..] \| \| \| \| \| \| \| \| \| 1. SusceptibilityCiprofloxacin 4 \| ⭘ S \| \| ⭘ I \| \| ⭘ R \| \| ⭘ Not done \| \| \| 1. MIC Ciprofloxacin 4 \| […………………………..] \| \| \| \| \| \| \| \| \| 1. SusceptibilityGentamicin 4 \| ⭘ S \| \| ⭘ I \| \| ⭘ R \| \| ⭘ Not done \| \| \| 1. MIC Gentamicin 4 \| […………………………..] \| \| \| \| \| \| \| \| \| 1. Susceptibitity Ampicillin 4 \| ⭘ S \| \| ⭘ I \| \| ⭘ R \| \| ⭘ Not done \| \| \| 1. MIC Ampicillin 4 \| […………………………..] \| \| \| \| \| \| \| \| \| 1. SusceptibilityGentmicin-Syn 4 \| ⭘ S \| \| ⭘ I \| \| ⭘ R \| \| ⭘ Not done \| \| \| 1. MIC Gentmicin-Syn 4 \| […………………………..] \| \| \| \| \| \| \| \| \| 1. SusceptibilityNitrofurantoin 4 \| ⭘ S \| \| ⭘ I \| \| ⭘ R \| \| ⭘ Not done \| \| \| 1. MIC Nitrofurantoin 4 \| […………………………..] \| \| \| \| \| \| \| \| |

| **LAB TEST RESULTS LAB (5 / 8)** |
| --- |
| **GRAM NEGATIVE BACTERIA** |
| 1. Sample 1 ⭘ Vaginal swab ⭘ Rectal swab ⭘ Other […………………….] 2. Isolation1 ⭘ Pos ⭘ Neg ⭘ Not done ⭘ Other […………………….] 3. Name of isolation 1 [……………………………………] 4. Date of isolation 1 [___\|___]/[___\|___]/[___\|___\|___\|___] (dd/mm/yyyy)  \| 1. Susceptibitity Ampicillin 1 \| ⭘ S \| ⭘ I \| ⭘ R \| ⭘ Not done \| \| --- \| --- \| --- \| --- \| --- \| \| 1. MIC Ampicillin 1 \| […………………………..] \| \| \| \| \| 1. Susceptibitity Cefazolin 1 \| ⭘ S \| ⭘ I \| ⭘ R \| ⭘ Not done \| \| 1. MIC Cefazolin 1 \| […………………………..] \| \| \| \| \| 1. Susceptibitity Gentamicin 1 \| ⭘ S \| ⭘ I \| ⭘ R \| ⭘ Not done \| \| 1. MIC Gentamicin 1 \| […………………………..] \| \| \| \| \| 1. Susceptibitity Amikacin 1 \| ⭘ S \| ⭘ I \| ⭘ R \| ⭘ Not done \| \| 1. MIC Amikacin 1 \| […………………………..] \| \| \| \| \| 1. SusceptibilityCeftriaxone 1 \| ⭘ S \| ⭘ I \| ⭘ R \| ⭘ Not done \| \| 1. MIC Ceftriaxone 1 \| […………………………..] \| \| \| \| \| 1. Susceptibitity Cefepime 1 \| ⭘ S \| ⭘ I \| ⭘ R \| ⭘ Not done \| \| 1. MIC Cefepime 1 \| […………………………..] \| \| \| \| \| 1. Susceptibitity Ertapenem 1 \| ⭘ S \| ⭘ I \| ⭘ R \| ⭘ Not done \| \| 1. MIC Ertapenem 1 \| […………………………..] \| \| \| \| \| 1. Susceptibitity Imipenem 1 \| ⭘ S \| ⭘ I \| ⭘ R \| ⭘ Not done \| \| 1. MIC Imipenem 1 \| […………………………..] \| \| \| \| \| 1. Susceptibitity Meropenem 1 \| ⭘ S \| ⭘ I \| ⭘ R \| ⭘ Not done \| \| 1. MIC Meropenem 1 \| […………………………..] \| \| \| \| \| 1. Susceptibitity Ampicillin-Sulbactam 1 \| ⭘ S \| ⭘ I \| ⭘ R \| ⭘ Not done \| \| 1. MIC Ampicillin-Sulbactam 1 \| […………………………..] \| \| \| \| \| 1. Susceptibitity Piperacillin-Tazobactam 1 \| ⭘ S \| ⭘ I \| ⭘ R \| ⭘ Not done \| \| 1. MIC Piperacillin-Tazobactam 1 \| […………………………..] \| \| \| \| \| 1. SusceptibilityTrimethoprim-Sulfamethoxazole 1 \| ⭘ S \| ⭘ I \| ⭘ R \| ⭘ Not done \| \| 1. MIC Trimethoprim-Sulfamethoxazole 1 \| […………………………..] \| \| \| \| \| 1. SusceptibilityCeftazidime –Avibactam 1 \| ⭘ S \| ⭘ I \| ⭘ R \| ⭘ Not done \| \| 1. MIC Ceftazidime –Avibactam 1 \| […………………………..] \| \| \| \| \| 1. SusceptibilityCiprofloxacin 1 \| ⭘ S \| ⭘ I \| ⭘ R \| ⭘ Not done \| \| 1. MIC Ciprofloxacin 1 \| […………………………..] \| \| \| \| \| 1. SusceptibilityTygecycline 1 \| ⭘ S \| ⭘ I \| ⭘ R \| ⭘ Not done \| \| 1. MIC Tygecycline 1 \| […………………………..] \| \| \| \| \| 1. SusceptibilityCefuroxime 1 \| ⭘ S \| ⭘ I \| ⭘ R \| ⭘ Not done \| \| 1. MIC Cefuroxime 1 \| […………………………..] \| \| \| \| \| 1. Độ nhạy cảm Levofloxacin 1 \| ⭘ S \| ⭘ I \| ⭘ R \| ⭘ Not done \| \| 1. MIC Levofloxacin 1 \| […………………………..] \| \| \| \| \| 1. Độ nhạy cảm Ceftazidime 1 \| ⭘ S \| ⭘ I \| ⭘ R \| ⭘ Not done \| \| 1. MIC Ceftazidime 1 \| […………………………..] \| \| \| \| \| 1. Độ nhạy cảm Minocycline 1 \| ⭘ S \| ⭘ I \| ⭘ R \| ⭘ Not done \| \| 1. MIC Minocycline 1 \| […………………………..] \| \| \| \| \| 1. Độ nhạy cảm Doxycycline 1 \| ⭘ S \| ⭘ I \| ⭘ R \| ⭘ Not done \| \| 1. MIC Doxycycline 1 \| […………………………..] \| \| \| \| \| 1. ESBL \| ⭘ Positive \| \| ⭘ Negative \| \| |

| **LAB TEST RESULTS LAB (6 / 8)** |
| --- |
| **GRAM NEGATIVE BACTERIA** |
| 1. Sample 2 ⭘ Vaginal swab ⭘ Rectal swab ⭘ Other […………………….] 2. Isolation2 ⭘ Pos ⭘ Neg ⭘ Not done ⭘ Other […………………….] 3. Name of isolation 2 [……………………………………] 4. Date of isolation 2 [___\|___]/[___\|___]/[___\|___\|___\|___] (dd/mm/yyyy)  \| 1. Susceptibitity Ampicillin 2 \| ⭘ S \| ⭘ I \| ⭘ R \| ⭘ Not done \| \| --- \| --- \| --- \| --- \| --- \| \| 1. MIC Ampicillin 2 \| […………………………..] \| \| \| \| \| 1. Susceptibitity Cefazolin 2 \| ⭘ S \| ⭘ I \| ⭘ R \| ⭘ Not done \| \| 1. MIC Cefazolin 2 \| […………………………..] \| \| \| \| \| 1. Susceptibitity Gentamicin 2 \| ⭘ S \| ⭘ I \| ⭘ R \| ⭘ Not done \| \| 1. MIC Gentamicin 2 \| […………………………..] \| \| \| \| \| 1. Susceptibitity Amikacin 2 \| ⭘ S \| ⭘ I \| ⭘ R \| ⭘ Not done \| \| 1. MIC Amikacin 2 \| […………………………..] \| \| \| \| \| 1. SusceptibilityCeftriaxone 2 \| ⭘ S \| ⭘ I \| ⭘ R \| ⭘ Not done \| \| 1. MIC Ceftriaxone 2 \| […………………………..] \| \| \| \| \| 1. Susceptibitity Cefepime 2 \| ⭘ S \| ⭘ I \| ⭘ R \| ⭘ Not done \| \| 1. MIC Cefepime 2 \| […………………………..] \| \| \| \| \| 1. Susceptibitity Ertapenem 2 \| ⭘ S \| ⭘ I \| ⭘ R \| ⭘ Not done \| \| 1. MIC Ertapenem 2 \| […………………………..] \| \| \| \| \| 1. Susceptibitity Imipenem 2 \| ⭘ S \| ⭘ I \| ⭘ R \| ⭘ Not done \| \| 1. MIC Imipenem 2 \| […………………………..] \| \| \| \| \| 1. Susceptibitity Meropenem 2 \| ⭘ S \| ⭘ I \| ⭘ R \| ⭘ Not done \| \| 1. MIC Meropenem 2 \| […………………………..] \| \| \| \| \| 1. Susceptibitity Ampicillin-Sulbactam 2 \| ⭘ S \| ⭘ I \| ⭘ R \| ⭘ Not done \| \| 1. MIC Ampicillin-Sulbactam 2 \| […………………………..] \| \| \| \| \| 1. Susceptibitity Piperacillin-Tazobactam 2 \| ⭘ S \| ⭘ I \| ⭘ R \| ⭘ Not done \| \| 1. MIC Piperacillin-Tazobactam 2 \| […………………………..] \| \| \| \| \| 1. SusceptibilityTrimethoprim-Sulfamethoxazole 2 \| ⭘ S \| ⭘ I \| ⭘ R \| ⭘ Not done \| \| 1. MIC Trimethoprim-Sulfamethoxazole 2 \| […………………………..] \| \| \| \| \| 1. SusceptibilityCeftazidime –Avibactam 2 \| ⭘ S \| ⭘ I \| ⭘ R \| ⭘ Not done \| \| 1. MIC Ceftazidime –Avibactam 2 \| […………………………..] \| \| \| \| \| 1. SusceptibilityCiprofloxacin 2 \| ⭘ S \| ⭘ I \| ⭘ R \| ⭘ Not done \| \| 1. MIC Ciprofloxacin 2 \| […………………………..] \| \| \| \| \| 1. SusceptibilityTygecycline 2 \| ⭘ S \| ⭘ I \| ⭘ R \| ⭘ Not done \| \| 1. MIC Tygecycline 2 \| […………………………..] \| \| \| \| \| 1. SusceptibilityCefuroxime 2 \| ⭘ S \| ⭘ I \| ⭘ R \| ⭘ Not done \| \| 1. MIC Cefuroxime 2 \| […………………………..] \| \| \| \| \| 1. Độ nhạy cảm Levofloxacin 2 \| ⭘ S \| ⭘ I \| ⭘ R \| ⭘ Not done \| \| 1. MIC Levofloxacin 2 \| […………………………..] \| \| \| \| \| 1. Độ nhạy cảm Ceftazidime 2 \| ⭘ S \| ⭘ I \| ⭘ R \| ⭘ Not done \| \| 1. MIC Ceftazidime 2 \| […………………………..] \| \| \| \| \| 1. Độ nhạy cảm Minocycline 2 \| ⭘ S \| ⭘ I \| ⭘ R \| ⭘ Not done \| \| 1. MIC Minocycline 2 \| […………………………..] \| \| \| \| \| 1. Độ nhạy cảm Doxycycline 2 \| ⭘ S \| ⭘ I \| ⭘ R \| ⭘ Not done \| \| 1. MIC Doxycycline 2 \| […………………………..] \| \| \| \| \| 1. ESBL \| ⭘ Positive \| \| ⭘ Negative \| \| |

| **LAB TEST RESULTS LAB (7 / 8)** |
| --- |
| **GRAM NEGATIVE BACTERIA** |
| 1. Sample 3 ⭘ Vaginal swab ⭘ Rectal swab ⭘ Other […………………….] 2. Isolation3 ⭘ Pos ⭘ Neg ⭘ Not done ⭘ Other […………………….] 3. Name of isolation 3 [……………………………………] 4. Date of isolation 3 [___\|___]/[___\|___]/[___\|___\|___\|___] (dd/mm/yyyy)  \| 1. Susceptibitity Ampicillin 3 \| ⭘ S \| ⭘ I \| ⭘ R \| ⭘ Not done \| \| --- \| --- \| --- \| --- \| --- \| \| 1. MIC Ampicillin 3 \| […………………………..] \| \| \| \| \| 1. Susceptibitity Cefazolin 3 \| ⭘ S \| ⭘ I \| ⭘ R \| ⭘ Not done \| \| 1. MIC Cefazolin 3 \| […………………………..] \| \| \| \| \| 1. Susceptibitity Gentamicin 3 \| ⭘ S \| ⭘ I \| ⭘ R \| ⭘ Not done \| \| 1. MIC Gentamicin 3 \| […………………………..] \| \| \| \| \| 1. Susceptibitity Amikacin 3 \| ⭘ S \| ⭘ I \| ⭘ R \| ⭘ Not done \| \| 1. MIC Amikacin 3 \| […………………………..] \| \| \| \| \| 1. SusceptibilityCeftriaxone 3 \| ⭘ S \| ⭘ I \| ⭘ R \| ⭘ Not done \| \| 1. MIC Ceftriaxone 3 \| […………………………..] \| \| \| \| \| 1. Susceptibitity Cefepime 3 \| ⭘ S \| ⭘ I \| ⭘ R \| ⭘ Not done \| \| 1. MIC Cefepime 3 \| […………………………..] \| \| \| \| \| 1. Susceptibitity Ertapenem 3 \| ⭘ S \| ⭘ I \| ⭘ R \| ⭘ Not done \| \| 1. MIC Ertapenem 3 \| […………………………..] \| \| \| \| \| 1. Susceptibitity Imipenem 3 \| ⭘ S \| ⭘ I \| ⭘ R \| ⭘ Not done \| \| 1. MIC Imipenem 3 \| […………………………..] \| \| \| \| \| 1. Susceptibitity Meropenem 3 \| ⭘ S \| ⭘ I \| ⭘ R \| ⭘ Not done \| \| 1. MIC Meropenem 3 \| […………………………..] \| \| \| \| \| 1. Susceptibitity Ampicillin-Sulbactam 3 \| ⭘ S \| ⭘ I \| ⭘ R \| ⭘ Not done \| \| 1. MIC Ampicillin-Sulbactam 3 \| […………………………..] \| \| \| \| \| 1. Susceptibitity Piperacillin-Tazobactam 3 \| ⭘ S \| ⭘ I \| ⭘ R \| ⭘ Not done \| \| 1. MIC Piperacillin-Tazobactam 3 \| […………………………..] \| \| \| \| \| 1. SusceptibilityTrimethoprim-Sulfamethoxazole 3 \| ⭘ S \| ⭘ I \| ⭘ R \| ⭘ Not done \| \| 1. MIC Trimethoprim-Sulfamethoxazole 3 \| […………………………..] \| \| \| \| \| 1. SusceptibilityCeftazidime –Avibactam 3 \| ⭘ S \| ⭘ I \| ⭘ R \| ⭘ Not done \| \| 1. MIC Ceftazidime –Avibactam 3 \| […………………………..] \| \| \| \| \| 1. SusceptibilityCiprofloxacin 3 \| ⭘ S \| ⭘ I \| ⭘ R \| ⭘ Not done \| \| 1. MIC Ciprofloxacin 3 \| […………………………..] \| \| \| \| \| 1. SusceptibilityTygecycline 3 \| ⭘ S \| ⭘ I \| ⭘ R \| ⭘ Not done \| \| 1. MIC Tygecycline 3 \| […………………………..] \| \| \| \| \| 1. SusceptibilityCefuroxime 3 \| ⭘ S \| ⭘ I \| ⭘ R \| ⭘ Not done \| \| 1. MIC Cefuroxime 3 \| […………………………..] \| \| \| \| \| 1. Độ nhạy cảm Levofloxacin 3 \| ⭘ S \| ⭘ I \| ⭘ R \| ⭘ Not done \| \| 1. MIC Levofloxacin 3 \| […………………………..] \| \| \| \| \| 1. Độ nhạy cảm Ceftazidime 3 \| ⭘ S \| ⭘ I \| ⭘ R \| ⭘ Not done \| \| 1. MIC Ceftazidime 3 \| […………………………..] \| \| \| \| \| 1. Độ nhạy cảm Minocycline 3 \| ⭘ S \| ⭘ I \| ⭘ R \| ⭘ Not done \| \| 1. MIC Minocycline 3 \| […………………………..] \| \| \| \| \| 1. Độ nhạy cảm Doxycycline 3 \| ⭘ S \| ⭘ I \| ⭘ R \| ⭘ Not done \| \| 1. MIC Doxycycline 3 \| […………………………..] \| \| \| \| \| 1. ESBL \| ⭘ Positive \| \| ⭘ Negative \| \| |

| **LAB TEST RESULTS LAB (8 / 8)** |
| --- |
| **GRAM NEGATIVE BACTERIA** |
| 1. Sample 4 ⭘ Vaginal swab ⭘ Rectal swab ⭘ Other […………………….] 2. Isolation3 ⭘ Pos ⭘ Neg ⭘ Not done ⭘ Other […………………….] 3. Name of isolation 3 [……………………………………] 4. Date of isolation 3 [___\|___]/[___\|___]/[___\|___\|___\|___] (dd/mm/yyyy)  \| 1. Susceptibitity Ampicillin 3 \| ⭘ S \| ⭘ I \| ⭘ R \| ⭘ Not done \| \| --- \| --- \| --- \| --- \| --- \| \| 1. MIC Ampicillin 3 \| […………………………..] \| \| \| \| \| 1. Susceptibitity Cefazolin 3 \| ⭘ S \| ⭘ I \| ⭘ R \| ⭘ Not done \| \| 1. MIC Cefazolin 3 \| […………………………..] \| \| \| \| \| 1. Susceptibitity Gentamicin 3 \| ⭘ S \| ⭘ I \| ⭘ R \| ⭘ Not done \| \| 1. MIC Gentamicin 3 \| […………………………..] \| \| \| \| \| 1. Susceptibitity Amikacin 3 \| ⭘ S \| ⭘ I \| ⭘ R \| ⭘ Not done \| \| 1. MIC Amikacin 3 \| […………………………..] \| \| \| \| \| 1. SusceptibilityCeftriaxone 3 \| ⭘ S \| ⭘ I \| ⭘ R \| ⭘ Not done \| \| 1. MIC Ceftriaxone 3 \| […………………………..] \| \| \| \| \| 1. Susceptibitity Cefepime 3 \| ⭘ S \| ⭘ I \| ⭘ R \| ⭘ Not done \| \| 1. MIC Cefepime 3 \| […………………………..] \| \| \| \| \| 1. Susceptibitity Ertapenem 3 \| ⭘ S \| ⭘ I \| ⭘ R \| ⭘ Not done \| \| 1. MIC Ertapenem 3 \| […………………………..] \| \| \| \| \| 1. Susceptibitity Imipenem 3 \| ⭘ S \| ⭘ I \| ⭘ R \| ⭘ Not done \| \| 1. MIC Imipenem 3 \| […………………………..] \| \| \| \| \| 1. Susceptibitity Meropenem 3 \| ⭘ S \| ⭘ I \| ⭘ R \| ⭘ Not done \| \| 1. MIC Meropenem 3 \| […………………………..] \| \| \| \| \| 1. Susceptibitity Ampicillin-Sulbactam 3 \| ⭘ S \| ⭘ I \| ⭘ R \| ⭘ Not done \| \| 1. MIC Ampicillin-Sulbactam 3 \| […………………………..] \| \| \| \| \| 1. Susceptibitity Piperacillin-Tazobactam 3 \| ⭘ S \| ⭘ I \| ⭘ R \| ⭘ Not done \| \| 1. MIC Piperacillin-Tazobactam 3 \| […………………………..] \| \| \| \| \| 1. SusceptibilityTrimethoprim-Sulfamethoxazole 3 \| ⭘ S \| ⭘ I \| ⭘ R \| ⭘ Not done \| \| 1. MIC Trimethoprim-Sulfamethoxazole 3 \| […………………………..] \| \| \| \| \| 1. SusceptibilityCeftazidime –Avibactam 3 \| ⭘ S \| ⭘ I \| ⭘ R \| ⭘ Not done \| \| 1. MIC Ceftazidime –Avibactam 3 \| […………………………..] \| \| \| \| \| 1. SusceptibilityCiprofloxacin 3 \| ⭘ S \| ⭘ I \| ⭘ R \| ⭘ Not done \| \| 1. MIC Ciprofloxacin 3 \| […………………………..] \| \| \| \| \| 1. SusceptibilityTygecycline 3 \| ⭘ S \| ⭘ I \| ⭘ R \| ⭘ Not done \| \| 1. MIC Tygecycline 3 \| […………………………..] \| \| \| \| \| 1. SusceptibilityCefuroxime 3 \| ⭘ S \| ⭘ I \| ⭘ R \| ⭘ Not done \| \| 1. MIC Cefuroxime 3 \| […………………………..] \| \| \| \| \| 1. Độ nhạy cảm Levofloxacin 3 \| ⭘ S \| ⭘ I \| ⭘ R \| ⭘ Not done \| \| 1. MIC Levofloxacin 3 \| […………………………..] \| \| \| \| \| 1. Độ nhạy cảm Ceftazidime 3 \| ⭘ S \| ⭘ I \| ⭘ R \| ⭘ Not done \| \| 1. MIC Ceftazidime 3 \| […………………………..] \| \| \| \| \| 1. Độ nhạy cảm Minocycline 3 \| ⭘ S \| ⭘ I \| ⭘ R \| ⭘ Not done \| \| 1. MIC Minocycline 3 \| […………………………..] \| \| \| \| \| 1. Độ nhạy cảm Doxycycline 3 \| ⭘ S \| ⭘ I \| ⭘ R \| ⭘ Not done \| \| 1. MIC Doxycycline 3 \| […………………………..] \| \| \| \| \| 1. ESBL \| ⭘ Positive \| \| ⭘ Negative \| \| |

| **FOLLOW UP** | |  |  | | | **FU (1 / 1)** | |  |
| --- | --- | --- | --- | --- | --- | --- | --- | --- |
| Study code  **[34EN]** | | Site code  [ \| \| ] | Patient code  [ \| \| ] | | | Innitials  [ \| \| \| \| \| ] | |  |
| Date contacted to patient: [ \| ]/[ \| ]/[ \| ] (dd /mm /yy ) | | | | | | | |  |
| As you are having a baby, we would like to know how you are feeling. Please mark “X” in the box next to the answer which comes closest to how you have felt in the **past 7 days** – not just how you feel today. | | | | | | | |  |
| In the past 7 days: | | | | | | | |  |
| **1. I have been able to to laugh and see the funny side of things** | | | | **6. Things have been getting on top of me** | | | |  |
| ⭘ | As much as I always could | | | ⭘ | Yes, most of the time I haven’t been able to cope | | |  |
| ⭘ | Not quite so much now | | | ⭘ | Yes, sometimes I haven’t been coping as well as usual | | |  |
| ⭘ | Definitely not so much now | | | ⭘ | No, most of the time I have coped quite well | | |  |
| ⭘ | Not at all | | | ⭘ | No, I have been coping as well as ever | | |  |
| **2. I have looked forward with enjoyment to things** | | | | **7. I have been so unhappy that I have had difficulty sleeping:** | | |  |  |
| ⭘ | As much as I ever did | | | ⭘ | Yes, most of the time |  |  |  |
| ⭘ | Rather less than I used to | | | ⭘ | Yes, sometimes |  |  |  |
| ⭘ | Definitely less than I used to | | | ⭘ | Not very often |  |  |  |
| ⭘ | Hardly at all | | | ⭘ | No, not at all |  |  |  |
| **3. I have blamed myself unnecessarily when things went wrong:** | | | | **8. I have felt sad or miserable** | | | |  |
| ⭘ | Yes, most of the time | | | ⭘ | Yes, most of the time |  |  |  |
| ⭘ | Yes, some of the time | | | ⭘ | Yes, quite often |  |  |  |
| ⭘ | Not very often | | | ⭘ | Not very often |  |  |  |
| ⭘ | No, never | | | ⭘ | No, not at all |  |  |  |
| **4. I have been anxious or worried for no good reason** | | | | **9. I have been so unhappy that I have been crying** | | | |  |
| ⭘ | No, not at all | | | ⭘ | Yes, most of the time |  |  |  |
| ⭘ | Hardly ever | | | ⭘ | Yes, quite often |  |  |  |
| ⭘ | Yes, sometimes | | | ⭘ | Only occasionally |  |  |  |
| ⭘ | Yes, very often | | | ⭘ | No, never |  |  |  |
| **5. I have felt scared or panicky for no very good reason** | | | | **10. The thought of harming myself has occurred to me** | | | |  |
| ⭘ | Yes, quite a lot | | | ⭘ | Yes, quite often |  |  |  |
| ⭘ | Yes, sometimes | | | ⭘ | Sometimes |  |  |  |
| ⭘ | No, not much | | | ⭘ | Hardly ever |  |  |  |
| ⭘ | No, not at all | | | ⭘ | Never |  |  |  |
| **Date Completed: Total score: / 30 Total Score for Question 10: / 3**  **Person Completing:** | | | | | | | |  |
