# Supplementary material for: Understanding maternal sepsis risk factors and bacterial etiology: A case control study protocol
Source: PLoS One. 2024 Jun 26;19(6):e0305411. doi: 10.1371/journal.pone.0305411 (PMC11207175; doi:10.1371/journal.pone.0305411)
Supplement: Attachment — Submitted filename: PONE-D-23-40494_Reviewer Comment.docx [file pone.0305411.s003.docx]

Understanding maternal sepsis risk factors and bacterial etiology: a case-control study protocol

**Reviewer Comments:**

**Keyword:**

Antimicrobial resistance should not be in the keyword as it’s not in line with title of the protocol

**Abstract:**

Line 30 contain was thus grammatical error. A protocol can’t contain was. Overall grammatical errors should be rewrite.

Line 43 primary and secondary outcome should be mentioned clearly in the abstract section

Overall aim and title conflict rigorously.

**Introduction:**

Line 56 LMICs should be showed a grammatical mistake.

Line 63, 64 should contain background data more to prove the statement as well.

Line 65 referencing should be corrected [7] [8]

More epidemiology on bacterial profile and AMR should be reflected in accordance with the study objective of the following concern study.

**Material and Methods:**

Line 82 how cases and controls will be selected not clear. If same age and gestation will be treated as either case or control? Line 93 conflict with it without defining pregnant of which gestational age.

Line 97 mode of delivery should be specified by NVD or CS

The Table -1, Obstetrically Modified SOFA score according to the reference [11] was not mentioned why respiratory parameter, coagulation and liver parameter was changed was not described and reference should be mentioned for the justified modification.

In line 117-118 how Bacterial etiology and AMR profile will be assessed through transmission dynamics or genomic analysis (16s r RNA extraction or shotgun metagenomics in which region v4 or any other should be mentioned clearly.

The overall outcome should be focused and specified according to the research question and hypothesis that lack the specified protocol.

Line 128, For cases vaginal sample and for control group vaginal and rectal swabs will be taken .Need justification.

In line 139, Outcomes will be censored at 42 days on which basis or reference ort justification needed.

In line 141, why “Conditional Logistic 142 regression models will be used to estimate the odds ratios of the associations between risk factors” –is not clear.

The statistical analysis plan also requires specified statistical tool or software to run the analysis is simply missing in this section.

Line 179-179, Sample size calculation should be mention as a particular section describing detail power of the test and proportions.

**Discussion:**

The discussion portion should be needed some comparison with others in the perspective of AMR and bacterial profile.

The Line 196 the second limitation of this study isn’t justified and it perhaps not the limitation regarding the comparative study.

The strength of the study lacks in the protocol version.

**Final Comments:**

**Major Revision required**
