# Supplementary material for: Understanding maternal sepsis risk factors and bacterial etiology: A case control study protocol
Source: PLoS One. 2024 Jun 26;19(6):e0305411. doi: 10.1371/journal.pone.0305411 (PMC11207175; doi:10.1371/journal.pone.0305411)
Supplement: Attachment — Submitted filename: Review Plosone-Le Hong Nhung final.docx [file pone.0305411.s004.docx]

Dear Authors,

I believe the paper has its merits and is potentially useful for researcher concerning study protocol. As far as we know, a study protocol is an essential part of a research project. It is a document that describes the study in detail, including the background, rationale, objectives, design, methodology, statistical considerations, and organization of a clinical research study. The protocol acts as a ‘manual’ for members of the research team to ensure everyone adheres to the methods outlined. As the study gets underway, it can then be used to monitor the study’s progress and evaluate its outcomes. Thus, the authors have clearly demonstrated all the norms of a study protocol in right template that PLOSONE requested.

Following, I would like to review the main sections such as Abstract, Introduction, Material and Methods, Discussion

**Abtract:** should included the study registration number where applicable

**Introduction:** It seems that authors also give the rationale for the study. However, the way of demonstration has not been cleared enough (What is a design hypothesis to test, what new evidence it is anticipated to provide). Besides, should refered to the appropriate previous literature.

**Material and Methods:** the manuscript demonstrate the aim, design and setting study, inclusion and exclusion criteria.

For the sample size: It is necessary to calculate the sample size according to the WHO formula for case-control study, to see that it is appropriate to select 100-200 (case-controls). The authors also show that characteristics of participants and how sample will be selected. However, the description of processes, interventions and comparisons should be demonstrated more clear. Besides, when and how the outcomes will be measured need adding.

Other issues met the standard like data management plan, safety considerations, the type of data and statistical analyses planned

**Discussion**

The authors also address issues involved in performing the study that are not covered in other section like limitations of study design, dissemination plans.
